# Unveiling the Printability and Performance of 3D Printed Ethylene-Vinyl Acetate Stretchable Foams

**DOI:** 10.1021/acsami.6c10093

**Published:** 2026-07-16

**Authors:** Nariman Rajabifar, Amir Ameli

**Affiliations:** Department of Plastics Engineering, 14710 University of Massachusetts Lowell, 1 University Avenue, Lowell, Massachusetts 01854, United States

**Keywords:** mechanical properties, structure−property relationships, EVA foam 3D printing, deformation mechanisms, advanced materials

## Abstract

The rise of additive manufacturing has pivoted the trajectory of advanced materials with intricate geometries and precise design. Although various materials have been adapted to 3D printing, developing printable microcellular foams remains in its infancy. Herein, for the first time, we report 3D printed ethylene-vinyl acetate (EVA) foams enabled by expandable microspheres (EMs). Expandable filaments of EVA/EMs containing 0–10 wt % EM result in a tailored density in the range of 0.903–0.224 g/cm^3^. The viable temperature spectrum relies upon EM content, where higher EM loading leads to a wider printing window. The foam density further exhibits an optimal correlation with temperature, reaching its minimum at 230 °C, independent of EM content. Microstructural analyses reveal uniform morphologies wherein the foam’s cell density increases and cell size slightly drops with EM loading. The decay rate of Young’s modulus and tensile strength with density reduction is significantly lower compared to conventional foams, which is attributed to the reinforcing effect of the EM shell material. Strain rate and print pattern dependency of mechanical properties are also discussed. While unfoamed EVA exhibits strain hardening upon cyclic compressive loading, the addition of EMs introduces a counteracting strain-softening behavior proportional to EM content. The thermal analysis furthermore depicts that EM leaves a negligible impact on the crystallinity of EVA. The findings of this work highlight the potential of 3D printed EVA foams as lightweight and tunable materials for various applications.

## Graphical abstract

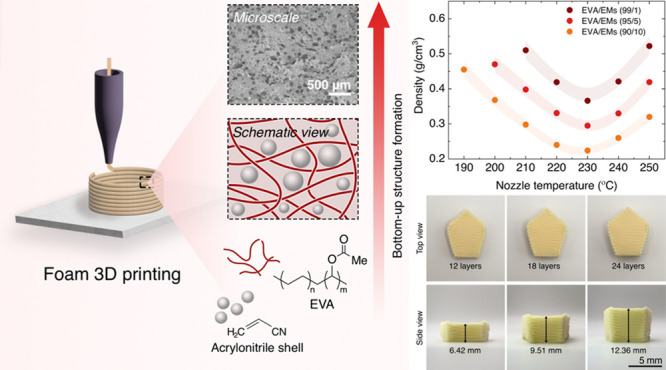

## 1 Introduction

Flexible and stretchable polymers have played an indispensable role in the realm of advanced materials over the past decades to meet the burgeoning need for numerous products featuring load-bearing attributes.^1–3^ Among an array of soft materials, ethylene-vinyl acetate (EVA) has drawn much attention due to its ease of processing, adequate toughness, chemical resistance, and flexibility.^4^ EVA is prevalently renowned for applications in footwear, packaging, sports equipment, and solar panel encapsulation. Imparting a foaming structure can induce a set of fresh purposes for EVA, rendering it more captivating for protective gear, mats, and buoyant materials for marine applications.^5,6^

In terms of manufacturing, fused filament fabrication (FFF), also known as material extrusion additive manufacturing, has reshaped polymer processing through on-demand and tool-free production of complex geometries following a computer-aided design (CAD) model.^7,8^ FFF operates by extruding a thermoplastic filament through a heated nozzle layer-by-layer. Precise spatial control, efficient utilization of materials, design freedom, and affordability are major advantages of FFF.

Within the FFF platform, different approaches have been earmarked to fabricate microcellular structures, including preprint foaming, postprint foaming, and in situ foaming.^9^ In the preprint foaming process, a cellular structure is introduced to the material during the filament fabrication phase, and the foamed filament is used to create printed parts.^10,11^ This approach is similar to syntactic foam printing in that the cell size, cell density, and foam density are predetermined, and there is no control over the foaming process during printing. In postprint foaming processes, the part is first printed and then separately foamed using an additional processing step.^12,13^ The printed part can be impregnated with high-pressure gas and foamed by using depressurization.^14,15^ The drawbacks of this method are (a) the need for an additional processing step, (b) limitations in control of the degree of foaming, and (c) shape/dimension control of the final foamed part.

Compared to preprint and postprint processes, in situ foaming has become more popular as it integrates foaming and printing steps into one process.^16,17^ Several types of blowing agents, including direct gas injection,^18^ chemical blowing agents,^19^ and expandable microspheres (EMs),^20^ have been utilized for in situ foam 3D printing. The direct gas injection method requires highly customized equipment to realize pressurized gas injection and foaming. It also requires a relatively high melt pressure and tight pressure drop control. Most chemical blowing agents also have adverse environmental effects. So far, it has been challenging to tightly control the foaming process and achieve uniform cellular morphologies by using direct gas injection and chemical blowing agents. The EM-assisted in situ foaming provides some promising solutions to these challenges. Overall, the rapid adoption of EMs lies primarily in the ease of the fabrication process of expandable filaments, superior control over foaming during printing, and the elimination of unnecessary steps or the need for additional equipment or customization.^21^ It has been shown that this approach provides the possibility of spatial control of density to fabricate functionally graded foam, an indication of great control over foaming during the printing process.^22^

EMs consist of a volatile hydrocarbon encapsulated in a thermoplastic shell. The shell is a copolymer, usually made of acrylonitrile, methacrylonitrile, and methyl methacrylate. The first two monomers are used due to their good gas barrier properties, and methyl methacrylate is used to modulate the glass transition temperature. Once heated above a certain temperature, the pressurized hydrocarbon core causes the softened shell to radially enlarge, providing cells of the cellular structure. EMs can expand up to 15 times their pre-expanded diameter.^23^ By streamlining the foaming process, these particles enable controlled foaming in a polymeric matrix and create a closed-cell structure. Depending on the EM content and the utilized printing parameters, the degree of foam expansion and consequently the density and physicomechanical properties of the 3D printed foams can be tailored to meet specific requirements.

EM-assisted foam 3D printing of several thermoplastic materials has been examined, including poly­(lactic acid) (PLA),^24^ polyethylene wax (PE),^25^ thermoplastic polyurethane (TPU),^26–28^ and acrylic (AC).^29^ These reports have documented the process success and some process-structure–property relationships of their own specific material systems. Moreover, recent studies have also demonstrated that foam 3D printing of elastomeric materials can enable graded, programmable, and functional structures. For instance, TPU-based foams have been used to realize origami structures with gradient stiffness to enable programmable deformation.^26^ Other reports have shown the feasibility of printing lightweight cellular geometries with tunable mechanical response.^20,28,29^ In addition, electrically conductive TPU/CNT foams have been recently printed with tunable density, conformality, and conductivity, making them potentially suitable for sensing applications in wearable and soft electronic devices.^30^ In this context, EVA-based printed foams can provide added value or a complementary platform to TPU systems, as EVA offers several advantages, including cost-effectiveness, reduced density, lower compression set, better cold-weather flexibility, and greater resistance to certain chemicals. Therefore, there is considerable interest in EVA printed foams.

While EVA has been extensively investigated in conventional manufacturing processes including injection molding, extrusion, and batch foaming, its adoption in FFF remains infacny due to its relatively poor melt strength, low softening point, high elasticity, and difficulties in printing process control.^31^ Because of their soft and elastomeric nature, EVA filaments are prone to buckling/jamming under the compressive forces required to feed the filament, which poses a notable challenge. Early attempts at FFF 3D printing of EVA have nevertheless shown some shape fidelity under optimized narrow printing parameters.^32,33^ EVA is required to remain molten long enough for interlayer bonding yet solidify quickly to support subsequent layers. These competing demands further complicate the 3D printing of elastic, semicrystalline EVA, which lacks the rapid stiffening behavior during cooling as seen in more rigid thermoplastics. Therefore, the FFF 3D printing of EVA foams has remained unexplored, demanding efforts to establish a solid understanding of the material formulation, printability, shape fidelity, and the resultant microstructural/morphological characteristics and physical attributes. Moreover, the mechanical performance of 3D printed EVA foams under repeated deformation or environmental exposure remains insufficiently understood. Thus, there is a chasm to develop EVA/EM formulations, the extrusion process of expandable filament, an optimized FFF process that balances the requirements for melt flow, interlayer adhesion, and foaming control, and ultimately assess the performance of the resultant foams.

This work aims to provide insights into the filament fabrication and foam 3D printing processes as well as the detailed mechanical and thermal characterizations of 3D printed EVA foams. The novelty of the current work lies in (a) the material combination of EMs and EVA, which is an inherently challenging material to 3D print, (b) exploiting dual roles of EMs as foaming agent and reinforcing filler, realizing EVA microcellular structures and improving their mechanical stiffness, printability, and shape fidelity, (c) process development and optimization for EVA-based expandable filament fabrication and its 3D printing, and (d) comprehensive characterizations to understand process-structure–property relationships. Microspheres serve as a reinforcing agent for the EVA filament, enhancing its stiffness and reducing the susceptibility to buckling while feeding. During in situ foaming, while they realize microcellular structure, the expanded EMs also act as a structural skeleton and improve the stability of the EVA melt, resulting in enhanced overall printability and a widened processing window. They also contribute to the stiffening of printed EVA foams. Our discussion entails the printability of EVA/EM foams at different nozzle temperatures and EM loadings to probe the relations between the blowing agent content and printing temperature with the density of the printed foams. The mechanical properties of the 3D printed objects were assessed under uniaxial tension and compression loadings. The strain rate behavior of the tensile properties, along with the compression set and hardness, is also discussed. The investigation of microstructure evolution reveals how cellular morphology changes with density. The thermal behavior of filaments and prints is analyzed to understand the role of EMs in the crystallization and decomposition temperatures of EVA.

## 2 Experimental Section

### 2.1 Filament Preparation and Foam 3D Printing

To prepare FFF filaments, EVA pellets (Cleanse 1821A, ρ = 0.938 g/cm^3^) and EM masterbatch (Expancel 980MB100 by Nouryon with an average particle size of 10–20 μm, according to the provider) were blended and extruded using a Collin E30P single-screw extruder with a diameter of 30 mm, a length to diameter ratio of *L*/*D* = 25, a screw speed of 8 rpm, and a residence time of about 11 min. The 980MB100 EM masterbatch contains 65 wt % hydrocarbon and 35 wt % EVA carrier. The 980MB100 EM masterbatch grade was selected because its activation temperature range matches the EVA melt processing temperature window and the polymer carrier is EVA, making it more compatible for mixing. The extruder zones from feeder to die were set at 80, 120, 145, and 145 °C, respectively. The fabricated filaments were collected on a Filabot spooler with a controlled diameter of 1.70 ± 0.05 mm after cooling in a water bath. The selected compositions of EVA/EMs were 100/0, 99/1, 95/5, and 90/10 wt %, where EVA weight ratios include the matrix EVA and masterbatch carrier EVA. All materials were dried at 40 °C for at least 4 h prior to melt mixing according to the manufacturer’s guidelines.

The CAD models (Figure S1) were created using Autodesk Fusion 360. G-codes for printing 3D models were generated using the IdeaMaker slicer. The fabricated EVA/EM filaments were loaded into an FFF 3D printer (Raise3D, Pro2 Series) with a 0.8 mm nozzle diameter and layer width and height at 0.8 and 0.5 mm, respectively. Platform temperature was fixed at 50 °C. A print speed of 10 mm/s was used for foams, while solid EVA was printable only at a speed of 4 mm/s or lower, and thus 4 mm/s was adapted for solid prints (Supporting Information - Section 1). A flow rate of 100% was used to print solid EVA, where 100% refers to the nominal feed-in volumetric flow rate, *Q* (approximated by bead cross-sectional area, 0.8 × 0.5 mm, multiplied by linear printhead speed, 4 mm/s) required for printing of typical solid print without under- or overextrusion. The 100% flow rate for solid EVA was thus about 1.6 mm^3^/s. For in situ foaming, the flow rate is deliberately chosen to be less than 100% so that a fraction of the nozzle output flow rate is supplied by foam expansion. Here, for EVA/EM foams, a 100% feed-in flow rate would be 4 mm^3^/s as the speed was 10 mm/s. However, a fractional flow rate of 60% (2.4 mm^3^/s) was used after optimization (Supporting Information - Section 1). Nozzle temperature and path direction were the main process variables. The former was varied in the range of 190–250 °C. For the latter, the tensile samples were printed with path directions parallel or perpendicular to the loading direction. No perimeters were used during printing, and the same layer direction was used for all layers within an object (Figure S1).

### 2.2 Material Characterizations

Scanning electron microscopy (SEM) of the solid and foamed samples was conducted using a JEOL JSM 6390 instrument with an accelerating voltage of 5 kV. The cross-sectional micrographs were acquired from the 3D printed bars, cryo-fractured perpendicular and parallel to the printing direction.

The density of the filaments and printed samples was calculated from the buoyancy force at room temperature using $\rho =\frac{m_{\mathrm{sa}}\times \rho_{l}}{\left(m_{\mathrm{sa}}-m_{\mathrm{sl}}\right)}$ , where *m*_sa_ is the sample mass measured in air, *m*_sl_ is the sample mass submerged in distilled water, and ρ_*l*_ is the density of water at the determined condition. The theoretical density was also computed using ρ = *w*_*i*_ρ_*i*_ + (1 – *w*_*i*_)­ρ_*j*_, where *w* is the weight fraction and *i* corresponds to the matrix (i.e., EVA). *j* denotes the unexpanded EM particles with ρ_*j*_ = 1.1 *g*/*cm* ^3^ based on the manufacturer’s datasheet. The density of EVA was 0.938 g/cm^3^ according to the manufacturer. The average cell diameter (D) and cell density (*N*_*A*_) were acquired from electron microscopy images by measuring at least 500 cells, using $D=\frac{\sum D_{i}n_{i}}{\sum n_{i}}$ and $N_{A}={\left(\frac{n_{i}}{A}\right)}^{3/2}\times \mathrm{ER}$ , where *D*_*i*_ is the cell diameter, *n*_*i*_ is the number of observed cells in the area under analysis (A), and ER is the foam expansion ratio.^34^ The latter element is calculated based on $\mathrm{ER}=\frac{\rho_{s}}{\rho_{f}}$ , where ρ_*s*_ is the density of solid and ρ_*f*_ is the density of foam.

The thermal decomposition behavior of the neat and foamable materials was observed on a Mettler Toledo thermogravimetric analyzer (TGA) from 45 to 600 °C with a 10 °C/min heating rate under nitrogen. The crystallization behavior of the materials before and after printing was measured on a Mettler Toledo differential scanning calorimeter (DSC) with a ramp sweep from −50 to 150 °C with a heating rate of 20 °C/min under nitrogen. A 9–10 mg sample was hermetically sealed in an aluminum pan and then exposed to a heat–cool-heat cycle. The crystallinity was calculated using $X_{c}=\frac{\Delta H_{f}}{\Delta H_{f}^{o}f_{\mathrm{EVA}}}\times 100;\;\Delta H_{f}^{o}=277.1(J/g)$ , where Δ*H*_*f*_ is the fusion enthalpy, *f* is the fraction of EVA content, and Δ*H*_*f*_^*o*^ is the pure fusion enthalpy of EVA. Three replications were conducted for thermal analysis experiments.

Tensile tests were carried out based on ASTM D638 using an Instron 5900 instrument equipped with a 5 kN load cell. Displacement rates of 10, 50, and 250 mm/s were used to evaluate the strain-rate effect. It is noted that these are not the strain rate values but produce a similar degree of variability in the strain rate. An optical extensometer was used to measure the sample’s displacement. Compressive properties of the 3D printed foams were also characterized using a 10 kN load cell mounted on an Instron 5966 machine in both monotonic and cyclic modes up to a strain of 50%, according to ASTM D3574. Cyclic compression was performed up to 50 consecutive loading–unloading cycles with a displacement rate of 10 mm/s to observe the effect of substantial loading on the printed foams. Tension and compression properties of the 3D printed foams were evaluated with five replications. Compression set behavior based on ASTM D3575 and hardness based on ASTM D2240 were tested for three replications under ambient conditions.

## 3 Results Discussion

### 3.1 3D Printing of EVA Foams

Table 1 presents the measured and theoretical densities of the EVA and EVA/EM filaments. The density of the fabricated solid EVA filament was relatively close to that reported by the manufacturer for EVA pellets, with less than 1% difference, which lies within the measurement error range. As the EM content was increased in the EVA/EM samples, the theoretical density slightly increased because the density of the EMs (1.1 g/cm^3^) was larger than EVA’s density (0.938 g/cm^3^). However, the measured density of EVA/EMs decreased with the EM content. This decrease is attributed to marginal expansion of the EMs during filament fabrication process. Nevertheless, the largest difference between the measured and theoretical densities was 5.4%, indicating a maximum of 5.4% density reduction during filament fabrication, which is negligible, compared to several fold expansions obtained during in situ foam printing, as discussed later. The overall suppression of EM expansion was further verified by examining the filament's SEM micrographs, as discussed later in this section. This density reduction was insignificant as the temperature during the filament fabrication remained sufficiently below the EM activation range.

**Table 1:** Density of the Fabricated EVA/EM Filaments before 3D Printing

composition	measured density (g/cm^3^)	theoretical density (g/cm^3^)
EVA	0.929 ± 0.012	0.938
EVA/EMs (99/1)	0.915 ± 0.016	0.939
EVA/EMs (95/5)	0.900 ± 0.047	0.943
EVA/EMs (90/10)	0.896 ± 0.031	0.948

The expansion of EMs occurs due to heat input during 3D printing after passing the EVA/EM filaments through the hot end, therefore, creating microcellular structures (Figure 1a). In foam 3D printing, the tailoring parameters inherent to FFF 3D printing, such as temperature and flow rate, can be employed to control the foaming process in situ, independent of pre- or postprocessing steps. The key to regulating the density in the prints using EMs lies in the applied heat energy, where exposure to high enough heat enables EMs to expand. As the temperature rises, the EM core first undergoes a phase change following the Clausius–Clapeyron equation and induces a migratory flux of gas molecules toward the shell.^35^ Upon further increase in temperature, the core is pressurized since the volume remains unchanged. Once the temperature reaches the onset activation range (*T*_*a*_ = 169–189 °C), which corresponds to the glass transition temperature of the shell copolymer, the core’s pressurized gas causes the softened shell to expand. As the expansion continues, the deforming shell undergoes strain hardening. This leads to deceleration of the shell stretch during expansion. At the same time, as the microsphere grows in volume, the internal pressure decreases. Moreover, because the shell wall thickness decreases upon expansion, some internal gas also starts to diffuse out. These counteractive phenomena eventually lead to an equilibrium state, known as the consolidation point, at which the internal pressure can no longer apply sufficient stress to deform the shell. The Mg­(OH)_2_ present in the shell material, as well as the EVA melt surrounding the shell, contributes to a reduction of the internal gas permeability and supports preserving the expanded microspheres.^36,37^ As an inorganic filler, Mg­(OH)_2_ not only yields a more torturous path for migrating gas molecules, thus expanding more, but also retards the thermal degradation of shells to improve the resistance to high heat exposure. Being a ternary copolymer with a random configuration, the high gas barrier property of acrylonitrile monomer helps to retain the gas inside the shell after inflation, similar to methacrylonitrile monomer.^38^ The latter monomer also increases the mechanical strength to achieve adequate performance under applied forces. The common third constituent is methyl methacrylate, which contributes to ease of processability and flexibility of the shell during expansion.

**Figure 1:**
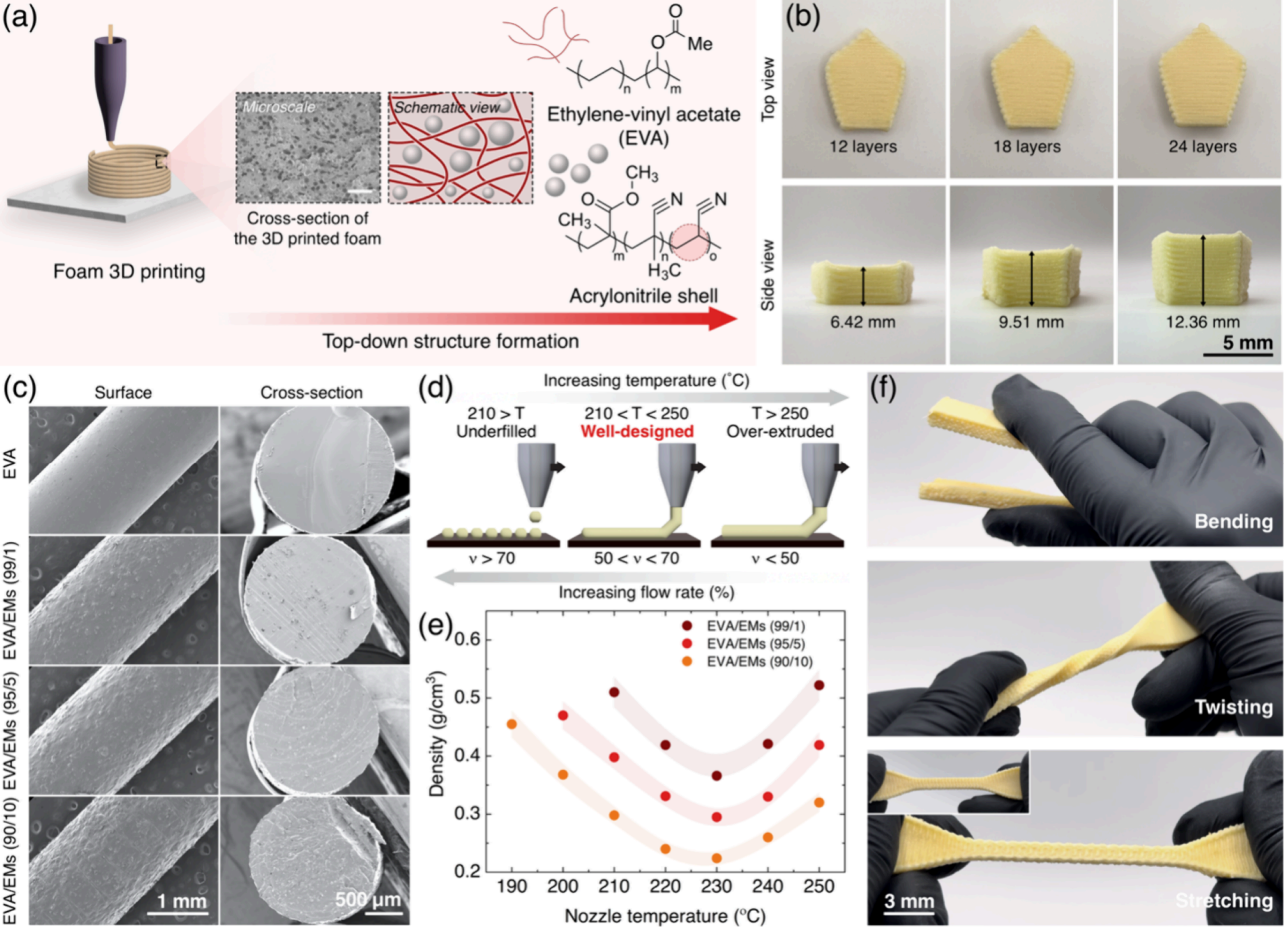
Foam 3D printing of EVA with tunable density. (a) Top-down structure formation of 3D printed foams. Scale bar: 500 μm. (b) Printability of EVA/EM foams shown by pentagon objects at different thicknesses. (c) Surface and cross-section SEM images of the EVA/EM foamable filaments. (d) Schematic representing the critical effects of temperature and flow rate values on the printability of EVA foams. (e) Viable range of densities for 3D printed EVA foams at various nozzle temperatures and EM contents. Shaded zones around the data points represent ± standard deviation. (f) Bendability, twisting, and stretchability of a 3D printed EVA/EMs foam at 95/5 composition.

Figure 1b shows the printability of EVA/EM foams by creating a pentagon object at different heights. The printability of EM-loaded filaments depends on several printing factors, including temperature and flow rate. Temperature is a major parameter affecting the quality, density, microstructure, and properties of the 3D printed foams.^39^

Figure 1c exhibits the SEM micrographs of the filaments’ surface and their cross-sectional view after cryo-fracturing. As seen, the cross sections of all fabricated filaments are tightly circular, showcasing the consistency of their diameter regardless of the EMs’ presence. In the case of neat EVA filament, no sign of EMs is observed. As the EM loading escalates from 0 to 10 wt %, an increase in the population of EM particles is obvious on both surface and cross-section micrographs. An EM size distribution of roughly 20–30 μm was observed in all EVA/EM filaments (Figure 1c), which agrees well with the manufacturer-provided pre-expanded size distribution. The existence of unexpanded EMs in the cross-section views corroborates that the applied heat and shear in the extrusion during filament fabrication were low enough to avoid expansion. Without employing the required heat for initiating the expansion in 3D printing, EMs are neither activated nor is the microcellular structure created. It is worth noting that very few EMs were found to be expanded, which explains the filaments’ minor density reduction (5.4%). This anomaly could be due to some localized shear/heat or extremely thin shell of these EMs, which could not withstand the internal gas pressure. Nonetheless, the influence of EMs on the surface roughness of the filaments is noteworthy, as they exhibit a textured appearance due to partial protrusion of EMs from the EVA surface.

Figure 1d displays the overall effect of temperature and flow rate on the EM expansion, and Figure S3 provides images of the samples printed at their various combinations. Figure S4 also summarizes the interplay between temperature and flow rate, providing the processable window and possible challenges and defects at the extremes of temperature and flow rate. By controlling the flow rate and print temperature, the shape fidelity and degree of expansion can be manipulated during foam 3D printing.^40^ The flow rate dictates how much of a material is fed to the printer's hot end, which affects both material volumetric input/output as well as the residence time, shear stress, and pressure. Lower flow rates provide less feed-in material but also slower flow, resulting in increased residence time, which, in turn, can increase foam expansion due to prolonged exposure to heat. The shear stress and pressure also decrease with a decrease in the flow rate, which could potentially ease the further EM expansion. However, extremely low flow rates can result in insufficient material throughput and shrinkage of expanded EMs due to long exposure times, both of which can yield underextrusion or droplet formation instead of a continuous bead during extrusion. The shrinkage of EMs due to long exposure occurs because the pressurized gas inside the microsphere finds ample time to diffuse out, causing internal pressure reduction. An example of shrunken and crumpled EMs can be seen in Figure S2. The onset of decay will also depend on other factors, including temperature and the EM shell thickness after expansion.

On the other hand, as the print temperature increases, the direct energy input to EMs goes up and causes faster and further activation of EMs. The temperature rise also lowers the melt viscosity. If the print temperature is below EM activation, the print would be essentially completed without any substantial foaming. As the temperature increases, the degree of foaming increases due to (a) high thermal energy available to the gasified blowing agent, (b) a more softened EM shell, and (c) reduced melt viscosity of the matrix. Higher temperatures provide higher gas pressure in the EM core while the resistance against shell expansion decreases due to more softened shells and the decreased internal stresses in the matrix due to lowered viscosity. However, by further increasing the print temperature beyond the EM’s activation window, the EMs experience significant expansion, and their shells become extremely thin, soft, and flexible. In this state, pressurized gas starts to diffuse out, causing shrinkage and thus a reduction in foaming degree. If the EM internal pressure is sufficiently low while the shell is soft and deformable, crumpling of EMs can also occur besides 3D shrinkage as the matrix is still flowing. This optimal temperature trend is clearly seen in Figure 1e. It should also be noted that the interplay between temperature and flow rate is very important and governs the process window for printability/foamability (Figure S4). For instance, extremely high flow rates, combined with high print temperatures, will provide excess material throughput and cause over extrusion along with possible foam shrinkage. However, if that is combined with too low temperature, the material may pass through the hot end so fast and so cold that it does not provide any chance for EMs to heat up sufficiently and expand, resulting in essentially unfoamed prints. If the flow rate is excessively low, combined with too high temperatures, most expanded EMs will eventually shrink and result in low quality foam along with under-extrusion. If that is combined with too low temperatures, no substantial foaming will occur, and the throughput will be so small, resulting in droplet formation instead of a continuous bead, rendering the material unprintable. Therefore, it is emphasized that the optimum shape fidelity and cell structure during foam 3D printing depend largely on the interplay between temperature and flow rate, and their combination within a certain range provides printable foams and governs the degree of expansion, as illustrated by the prints made using their various combinations in Figure S3. It is also worth noting that the EM activation occurs under transient thermal and pressure fields along and across the nozzle rather than at a steady state. However, no identifiable gradient in the resultant morphology was observed here.

As the most critical parameter in microcellular materials, density is driven by the extent of EM expansion during foaming. It was found that the low-end extremum of density reduction falls at 230 °C, where the least density for a 10 wt % EM-loaded filament stands at 0.224 g/cm^3^ (Figure 1e). This behavior, ubiquitous amid all compositions, unveils that the maximum expansion of the microspheres occurs around 230 °C, aligned with the manufacturer’s data. Beyond this point, the density reduction descends due to the rupture of the shell and the demolition of gaseous cells or their shrinkage due to gas loss through extremely thinned shells.^41^

At a given nozzle flow rate (60% here), the nozzle temperature range at which a printable foam was obtained depended on the EVA/EM composition. The printing temperature as low as 190 °C was sufficient to obtain a printable foam with a 90/10 composition, whereas the 95/5 composition started to exhibit printability at a higher temperature, i.e., 200 °C. This was further increased to 210 °C for the composition of 99/1. At print temperatures lower than these minimums, the polymer extrusion became notably intermittent, resulting in the formation of discrete, sputtered paths. This was attributed to inadequate melt throughput because of insufficient volume expansion, such that the prescribed partial flow rate of 60% together with minimal foam expansion did not provide enough material throughput and in-nozzle pressure build-up to fulfill the continuous extrusion of path volume as demanded by the design. In extrusion-based 3D printing, a steady flow is maintained only if a minimum level of pressure is preserved inside the nozzle. As the pressure decreases, the shear stress applied to the flowing melt decreases.^42^ When the pressure drops below a minimum, the material tends to come out as droplets rather than a continuous path. In this regime, the shear stresses are so low that the melt surface tension dominates the shear force in the extrudate, prompting droplets rather than consistent flow. Moreover, the droplet form reduces the contact area of the depositing melt and substrate and makes the wetting and adhesion poorer, relative to the case of a continuous bead. Therefore, the desirable, continuous print paths did not form, and the samples were deemed unprintable at these low temperatures. As the temperature increased beyond the minimum for each composition, due to sufficient foaming and pressure build-up, the shear forces prevailed over surface tension forces, and continuous flow was achieved. The decrease in minimum nozzle temperature with an increase in the EM content (Figure 1e) can also be explained by the fact that a greater number of EMs can provide a sufficient throughput increase and pressure build-up at a smaller expansion of each EM, which is attainable at lower temperatures.

Figure 1f shows the typical bending, twisting, and stretching capabilities of the 3D printed EVA/EM samples for the EVA/EM 95/5 composition. The integration of EMs has shown potential to enhance the flexibility of the matrix, aligning with the principles governing foam structures.^43^ The introduction of microcellular vacancies disrupts the continuity of the material, easing the deformability.

### 3.2 Cellular Structure of 3D Printed Foams

The overall quality of 3D printed foams depends on the quality of both surface and internal structure, which are both examined in Figure 2. As shown in Figure 2a, the edge view micrographs reveal that the layer height remained similar between solid and foamed samples, even though foamed samples were printed with a partial flow rate of 60%, as opposed to 100% for the solid case. Moreover, microspheres are also clearly observable on the surface of the foamed samples, a clear contrast to that of solid EVA. Looking at the face view row in this figure, the SEM micrographs reveal the path shape where the print direction reverses, and the path print direction changes by 180°. These micrographs indicate that the EMs were contained within the EVA matrix and expanded during printing without causing any path rupturing or physical integrity decay, even under these severe changes in the deposition direction.

**Figure 2:**
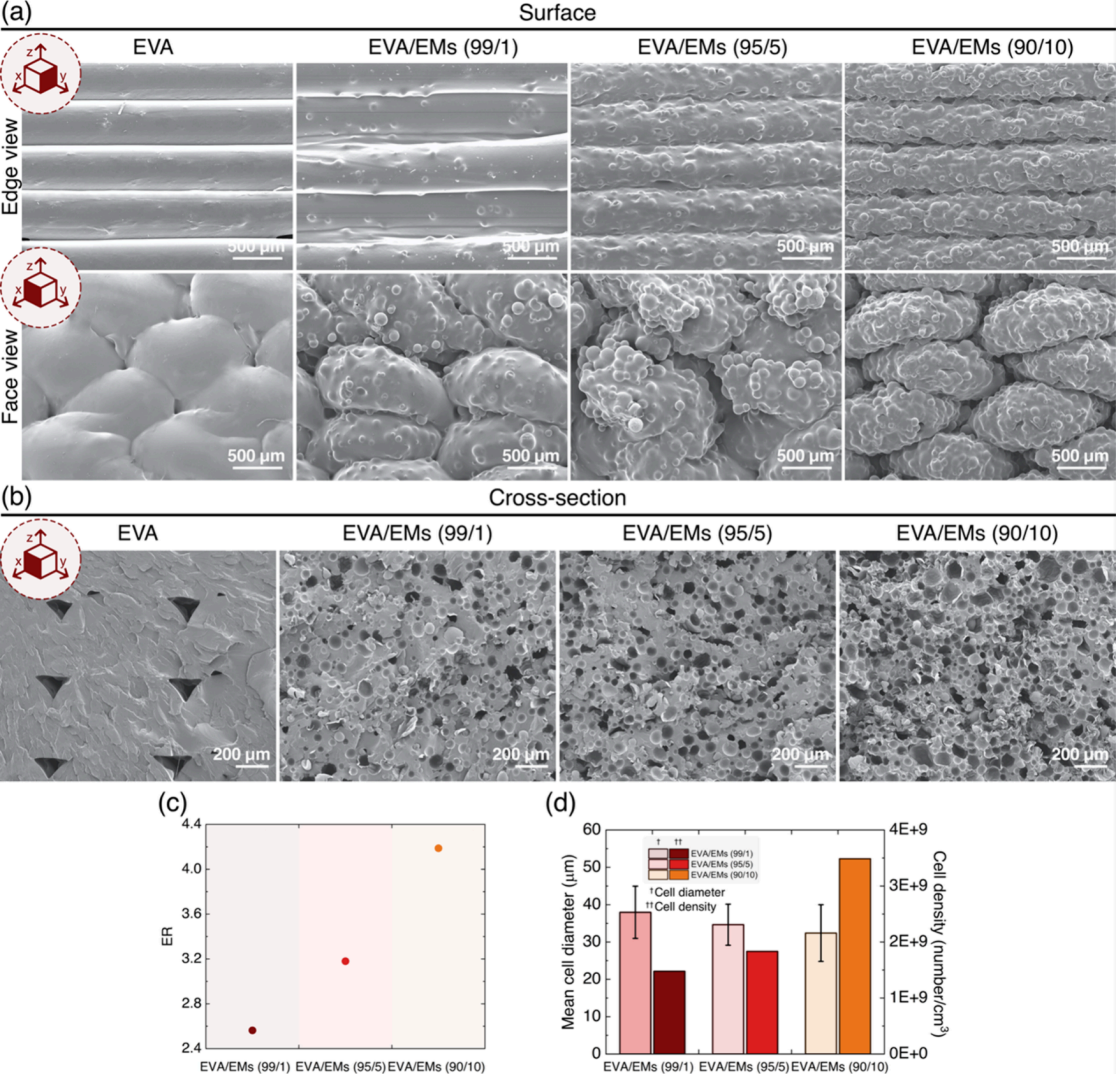
Cellular structure of the 3D printed foams. (a) SEM micrographs of the EVA and EVA/EM foam surfaces on edge and face views. (b) Cross-sectional micrographs of the 3D printed EVA and EVA/EM foams. In (a, b), *x* and z axes are the print and build directions, respectively. (c) Variation of expansion ratio (ER) with the EM content. (d) Variation of mean cell diameter and cell density with the EM content. The results are for EVA/EM foams printed at a nozzle temperature of 230°C. The error bars show ±1 standard deviation.

The cross-sectional SEM images of the printed samples reveal the microstructure and cellular morphology (Figure 2b). The micrographs show that the vacancies were relatively uniformly distributed within the EVA matrix, with a greater cell density at a higher EM content. As shown earlier, the printability of EVA foams is enhanced upon proper selection of the combination of temperature and flow rate that provides sufficient foam expansion. It is believed that the volumetric expansion in the deposited material contributes to the disappearance of the interlayer gaps that usually occur during the printing of a solid, unfoamed material. This is clearly observed by contrasting the SEM micrographs of Figure 2b. Another positive effect of foaming on printability is the reduction of the shrinkage and, consequently, the curl of the EVA upon addition of EMs. As the cooling starts, at higher temperatures where the EM shell is still soft, the internal pressure of EMs is transferable to the matrix and provides an overall internal pressure, causing a reduction in free shrinkage of the EVA matrix. As the cooling continues, the EM shell falls below its glass-transition temperature (i.e., the minimum activation temperature of EMs) and rigidifies. These rigid microspheres act as a scaffold and still result in a reduction in free shrinkage and warpage of the EVA matrix as it cools.

Figure 2c highlights the increase in ER by adding more EM content into the EVA samples, all printed under the same processing conditions (nozzle temperature of 230 °C and flow rate of 60%), in which 4.2 marks the maximum ER, corresponding to 10 wt % EM loading. The major drive for the ER increase from 99/1 to 90/10 samples is the cell density, which rises consistently with more EM content. As depicted in Figure 2d, the cell density increases by about 5 times with an increase of EM content from 1 to 10 wt %. Noting that the expansion ratio of 90/10 is nearly 2-fold for the 99/1 sample (Figure 2c), the ratio between the “nucleation cell densities” of 90/10 and 99/1 samples comes to about 10 (5 multiplied by 2), which agrees well with the fact that the 90/10 sample contained ten times more EM compared to 99/1. It is noted that the nucleation cell density refers to the number of cells before any foam expansion, which in this case should correspond to the number of EMs in the filament before expansion. Moreover, it is interesting to note that the cell size also varied with a change in the EM content, although with a much gentler rate. As the mean cell diameter of Figure 2d illustrates, the cell size was slightly reduced with an increase in the EM content. This behavior has previously been reported for 3D printed PLA foams, and it is attributed to the predetermined geometrical confinements and pressure levels applied by the nozzle tip to the exiting foam material.^41^ With an increase in the number of EMs, volumetric fulfillment is achieved by a smaller expansion of each EM before external factors, such as cooling and nozzle pressure, prevent further expansion.

### 3.3 Mechanical Behavior of 3D Printed EVA Foams

To understand and analyze the mechanical behavior of the printed foams, several evaluations were conducted including tensile, compression, hardness, and recovery (compression set) tests. The tensile tests were also conducted at various displacement rates of 10, 50, and 250 mm/s to probe the effect of the loading rate. In addition, the tensile samples were printed in two different directions, that is, the paths were printed parallel and perpendicular to the sample loading direction, to quantify the mechanical performance in the longitudinal and transverse directions. These samples were referred to as parallel prints and perpendicular prints hereafter.

Figure 3a,b exhibits the representative stress–strain curves of the 3D printed samples, tested under tension (displacement rate, ε̇ = 50 mm/s) and compression (ε̇ = 10 mm/s), respectively. In both tension and compression cases, as the EM content increased from 0 to 10 wt %, the strain energy (area under the stress–strain curve) declined. With an increase in the EM content, the bulk density of the samples lowered, and consequently, the solid fraction of the specimen that can tolerate and dissipate the applied external load was declined, resulting in a reduced strain energy.^44^ In addition to the proportional strain energy reduction with the density change, the applied stresses likely accumulate around the hollow content (cells), creating stress concentration locations and promoting easier crack initiation and propagation.^45^ The less pronounced plastic deformation in the foam samples, compared to the solid EVA sample (Figure 3a), indicates the action of such stress raisers. This causes further strain energy losses.

**Figure 3:**
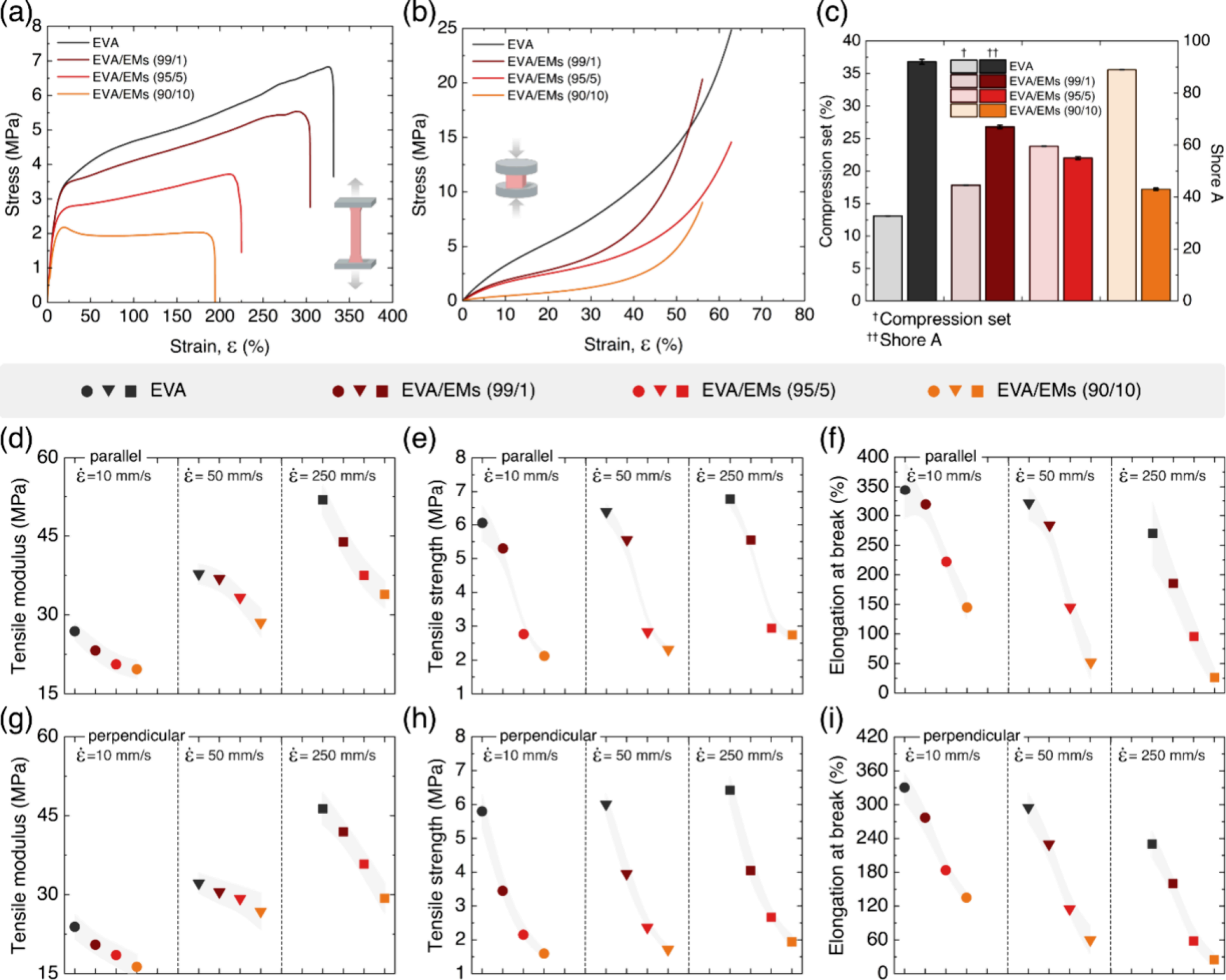
Mechanical behavior of 3D printed foams. (a) Representative tensile stress–strain curves of parallel-printed foams at a displacement rate of ε̇ = 50 mm/s. (b) Representative compression stress–strain curves at ε̇ = 10 mm/s. (c) Measured compression set and hardness (Shore A) of 3D printed EVA and EVA/EM foams. Tensile modulus, strength, and elongation at break for (d–f) parallel prints and (g–i) perpendicular prints. The density values are 0.903, 0.366, 0.295, and 0.224 g/cm^3^ for EVA and EVA/EMs of 99/1, 95/5, 90/10, respectively. The *x*-axis in parts (d–i) is the variation of EM content from 0 to 10 wt % from left to right. The error bars in (c) and the shaded zones around the data points in (d–i) represent ±1 standard deviation.

Figure 3c depicts the evolution of compression set and hardness in the printed samples as the EM content varies from 0 to 10 wt %. With the integration of more EM content, the compression set climbs relatively proportionally from nearly 13.08% of EVA to about 35.57% of 90/10 EVA/EMs. This behavior is attributed to more permanent and plastic deformation of the EM shell copolymer as opposed to more reversible deformation of the EVA matrix.^46^ The EM shell copolymer is in its glassy state at room temperature, unlike EVA, and the application of 50% strain for an extended period of 24 h imposes significant irreversible deformation through which a fraction of the applied energy is dissipated. Thus, upon the removal of the external load, the specimens consisting of EMs recover less. Moreover, as seen in Figure 3c, the printed samples’ hardness consistently decreases from nearly 92 to 43 shore A with an increase in the EM content from 0 to 10 wt %. This trend is in good agreement with the evolution of the sample’s stiffness as a function of EM content, as discussed later. Such behavior indicates that the hardness is primarily governed by the bulk density of the material, and as the density is decreased by adding more EM content, the solid fraction and structural support decline, and thus the sample becomes easier to indent. It is worth noting that the acrylonitrile-based copolymer shell is believed to be harder than EVA at room temperature; however, this material exists in the system as an extremely thin shell (usually submicrometer). It is well-known that the bending stiffness scales with the cube of thickness, such that very thin layers of even stiff polymers can exhibit effective flexibility and compliance. Consequently, the thin EM shell is unlikely to have substantial resistance against bending during the hardness test.

Figure 3d–f exhibits the tensile modulus, strength, and strain at break of parallel prints having various EM contents, tested at three displacement rates of 10, 50, and 250 mm/s, respectively. Similarly, Figure 3g–i depicts the same properties for perpendicular prints. With an increase in the EM content, the tensile modulus, strength, and strain at break all declined. Such a trend remains unaltered among parallel and perpendicular printing, as well as various displacement rates. However, the rate of the drop in these properties deviates from conventional foams fabricated with direct incorporation of physical or chemical blowing agents. The density dependency of foam’s modulus and strength is in general modeled using a power-law relationship (*Property* = *k*ρ^*n*^; *k*: *constant*, *n*: *power* – *law exponen* *t*) where n varies between 1 and 1.5 for closed-cell foams. A higher *n* value indicates a faster decay of the property with density reduction. Examining the modulus and strength dependencies of the EVA/EM foams reveals that *n* is significantly smaller than unity, indicating that the modulus and strength do not decay as quickly as that for conventional foams. For instance, the density drops 4.03 times from the solid EVA sample to the EVA/EM (90/10) sample, while the modulus and strength drop only about 1.36 and 2.85 in the case of parallel samples tested with a displacement rate of 10 mm/s. Such a great preservation of modulus and strength is attributed to the reinforcing effect of the EM shell material.^47,48^ As the EM shell is an acrylonitrile-based copolymer, it exhibits a higher modulus and strength, at least several folds, compared to the EVA matrix. These EVA/EM foams are essentially composite foams, where the EM shell material acts as a reinforcement and the EM core provides the cellular structure. Similar reinforcement effects have been reported in syntactic foams and micro balloon-filled polymer foams, where rigid or semirigid cell shells contribute to load transfer and delay cell-wall buckling, leading to higher modulus retention than what is expected from density reduction alone.^47–49^ In this work, the acrylonitrile-based copolymer shell of the EMs in EVA/EM foams plays a comparable reinforcing role.

When compared to conventionally manufactured closed-cell EVA foams at similar density, the 3D printed EVA/EM foams exhibit a distinct mechanical behavior.^50–52^ For example, the EVA/EM (90/10) foam with a density of 0.224 g/cm^3^ shows tensile strength comparable to that of commercial cross-linked EVA foam with a density of 200–235 kg/m^3^, but lower elongation at break and a higher compression set. In compression, the printed foam shows higher stress at 25% strain than the corresponding conventional EVA foam benchmark, suggesting enhanced large-strain compressive resistance, which can be attributed to the reinforcing contribution of the EM shells and possibly the printed architecture. It should be noted that as the exact EVA grade, vinyl acetate content, cross-linking state, and foaming formulation used in the literature are not identical to those used in this study, this comparison may be interpreted with caution.

Moreover, in both parallel and perpendicular cases, upon increasing the displacement rate, solid EVA and foamed EVA/EM samples all showed a growing trend in modulus and strength, which is due to the viscoelastic nature of polymers.^53^ The modulus of the solid 3D printed EVA increased nearly 2-fold from 10 to 250 mm/s of displacement rate, alongside an 11.58% increment in the tensile strength. Such a trend remained ubiquitous for foams as well. For example, these improvements for EVA/EMs (95/5) were 45.08 and 6.44% in the modulus and tensile strength, respectively. As the strain rate increases, the deformation time shortens, which provides less time for the polymer chains to relax, thus attenuating the viscous component and intensifying the elastic component of the viscoelastic behavior. Thus, the material behaves stiffer and stronger. Moreover, due to this time confinement and less molecular relaxation, the chains’ capability to slide past one another and undergo extended deformation reduces, and thus elongation at break decreases.

The parallel and perpendicular samples of solid EVA exhibited relatively similar moduli, strengths, and elongation at break values at the same strain rates. Although it has been reported that perpendicular samples show inferior performance relative to the parallel case,^54,55^ relatively similar performances of the two here indicate that a strong layer-to-layer adhesion was obtained between printed paths during the printing of EVA. However, this similarity was not maintained at such a high level with the introduction of foaming. Compared to those of parallel counterparts, the modulus and strength of perpendicular samples showed greater sensitivity to foaming and dropped with a slightly steeper rate with an increase in the EM content. The elongation at break of parallel and perpendicular samples, however, exhibited a somewhat similar dependence on the EM content. Nevertheless, the fairly similar values of strength and elongation at break in both printing directions indicate that the various degrees of foaming did not negatively affect the bonding between paths and can be viewed as a significant indication of the good printability of EVA/EM foams.

Figure 4a–d shows the cyclic compressive stress–strain of the 3D printed foams. In all the foamed samples, three distinct deformation zones are explicit in loading curves, including (I) a Hook's law regime in which the elastic bending of cell walls dominates the linear elasticity; (II) a plateau regime where buckling of the cell walls and energy absorption of the foam are determined; and (III) a densification regime in which frictional contact of encapsulated cells occurs.^56,57^ Starting with solid EVA, the onset of Hookean divergence falls before 15% strain, and plastic deformation is observed afterward. Because no voids and cell walls exist to buckle (zone II in foams), the stress continuously rises with the strain until the loading completes at 50% strain. Moving toward 3D printed foams reveals that the mechanical response under dynamic compressive stress transitions from viscoelastic to energy dissipation dominated by cellular collapse. As seen in Figure 4b–d, in the foamed samples, the stress first rises gradually at low strain levels, reflecting the initial elastic deformation of the material, although this zone appears to have a smaller strain range, compared to the EVA case. As strain progresses, the material undergoes a transition into zone II, where the stress increases more slowly with strain, indicating the onset of cell collapse. By further straining beyond 30–35%, the stress starts to rise more rapidly and nonlinearly, representing the compaction of the cells. In all the solid EVA and foamed EVA/EM cases, the unloading curve showed noticeable hysteresis in the first cycle, indicating energy dissipation due to internal molecular rearrangements and irreversible deformation in the polymer chains.^58,59^ For the first cycle unloading, the mean zero-stress strain values (set points) were recorded as 25.0 ± 5.19, 35.1 ± 2.53, 27.7 ± 2.12, and 39.5 ± 1.91% for EVA, EVA/EMs (99/1), EVA/EMs (95/5), EVA/EMs (90/10), respectively, indicating that the unloading curves failed to return to the zero-strain state, implying permanent deformation.^60^ Overall, the permanent deformation was greater in EVA/EM foams than in solid EVA samples, although no monotonic trend was observed with increasing EM content, indicating the need for further investigation. This additional set, in the presence of EMs, could be attributed to the severe irreversible deformation of EM shells or the full collapse of some microspheres, as discussed previously.

**Figure 4:**
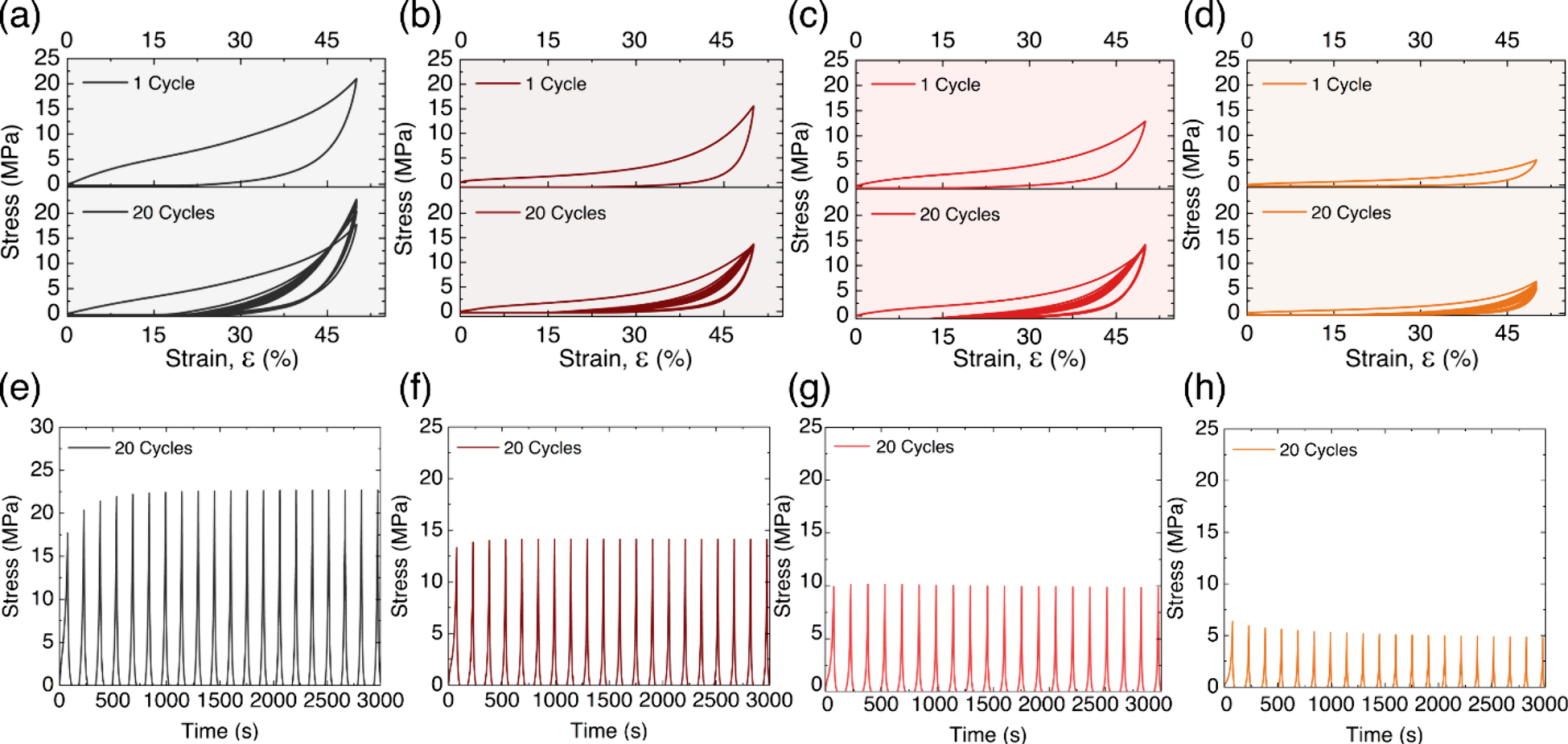
Cyclic compressive behavior of 3D printed EVA and EVA/EM foams. Representative stress–strain curves for (a) EVA, (b) EVA/EMs (99/1), (c) EVA/EMs (95/5), and (d) EVA/EMs (90/10). (e)–(h). The peak stress evolution with cycles up to 20 cycles for (e) EVA, (f) EVA/EMs (99/1), (g) EVA/EMs (95/5), and (h) EVA/EMs (90/10).

Figure 4e–h shows the evolution of peak stress (at 50% strain) with the cycle number up to 20 cycles for all of the samples. An interesting transition was observed for the peak stress evolution with the degree of foaming. In the early cycles, the peak stress rises with the cycle number in the case of solid EVA. This behavior is attenuated as foaming is introduced and eventually reversed such that a minimal rise in peak stress is observed for the case of EVA/EMs (99/1). With the increase of EM content to 5 wt % (95/5 case), the peak stress remains almost unchanged with the cycle number. Further addition of EM content to 10 wt % ultimately causes a reduction of peak stress with the cycle number. This observation could be attributed to the occurrence of two competing mechanisms: one contributing to the cyclic strain hardening of EVA chains and the other causing strain softening due to the presence of EMs. Due to the variation in the hysteresis effect with each cycle, accumulated residual strain, molecular rearrangement in the early cycles, and shrinking of free volume, especially in the case of printed materials, higher stresses are required to reach the same strain level after each cycle until a mechanical equilibrium is reached. On the other hand, under significant deformations (50% strain), the EMs present within the EVA can undergo interfacial debonding, cracking, and severe permanent deformation or cause localized yielding of EVA near the interface. All these factors can reduce the load-bearing capability of the EVA/EM foams at subsequent loading and thus cause strain softening. As the EM content increases, this effect is also signified. These two strain-induced mechanisms are counteractive, and they seem to balance each other out in the 95/5 case, where the peak stress remains nearly unchanged.

By the fiftieth cycle (Figure S5), the stress–strain curves have stabilized to a degree for all of the samples, with a less pronounced change in the peak stress and the hysteresis loop, compared to earlier cycles, showing that the samples have reached a quasi-equilibrium mechanical state.^61^ The hysteresis loop persists, albeit a little reduced compared to the initial cycles. The material may still be undergoing steady, incremental changes in mechanical behavior, but the rate of degradation has significantly diminished.^51^

### 3.4 Thermal Analysis of 3D Printed Foams

The TGA graphs of the EVA and EVA/EM filament samples, along with their first derivatives, are presented in Figure 5a,b and tabulated in Table 2. Three distinct temperature zones were identified, depicted as zone 1, zone 2, and zone 3 in Figure 5b. The residual mass at the end of zone 1 (∼220 to 290 °C) declined with an increase in the EM content, which is also observable in the DTG curves of Figure 5b inset. This mass loss is attributed to the release of pressurized hydrocarbons through the EM shell, causing a loss of up to a maximum of about 5% for the sample containing 10 wt % EM. Upon increasing the temperature, the two-step weight loss of EVA was observed, such that the deacetylation of vinyl acetate initially took place between 305 and 386 °C (zone 2), followed by chain scission of the polymer backbone at the last temperature range (zone 3).^52^ The significant weight loss, finished at ∼490–500 °C (*T*_3,endset_ in Table 2), reflects the completion of the EVA degradation. The addition of EMs shifted this temperature slightly to higher values. As seen in Table 2, the residual mass at the end of the test increases with an increase in the EM content, which could be due to the presence of inorganic additives in the EM shell.

**Figure 5:**
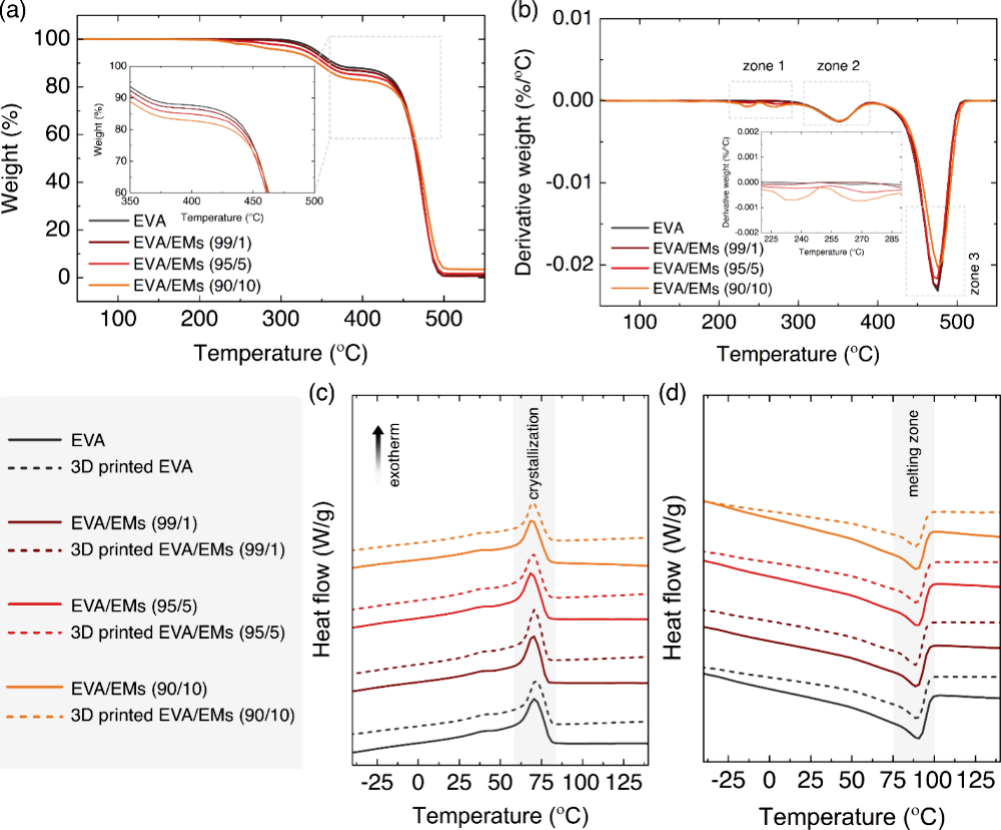
Thermal behavior of EVA and EVA/EMs. (a) Representative TGA curves of EVA and EVA/EM filaments. (b) Derivative of TGA curves for EVA and EVA/EM filaments. (c) Representative cooling cycle of the filaments and 3D printed objects. (d) Representative second heating cycle of the filaments and the first heating cycle of 3D printed objects. The inset in part (b) is a magnified look at zone 1.

**Table 2:** TGA and DTG Data of Zone 3 for EVA and EVA/EMs

	TGA		DTG (zone 3)		
specimen	residual mass at the end of Zone 1 (%)	char content (%)	*T*_3, onset_ (°C)	*T*_3, max_ (°C)	*T*_3, endset_ (°C)
EVA	87.52	0.22	443.15	475.50	493.84
EVA/EMs (99/1)	86.47	0.46	454.79	472.67	495.07
EVA/EMs (95/5)	85.03	1.27	458.23	471.33	495.55
EVA/EMs (90/10)	82.64	3.49	444.34	477.50	498.73

Figure 5c,d exhibits the DSC graphs of solid EVA and EVA/EM filaments and their 3D printed samples, and Table 3 provides a summary of the data. The changes in the peak melting temperatures for melting (*T*_m_) and crystallization (*T*_c_) were marginal with the addition of EM. Based on the data from the second heating of the filament samples, upon adding EMs, crystallinity slightly dropped as EMs physically disrupt the regular packing of EVA chains.^62^ In other words, compared to neat EVA, where the chains had the freedom to move and orient, the presence of solid-like microspheres during the crystallization of EVA disrupted the obstacle-free molecular motion and orientation of EVA, thus lowering the crystallinity. It is noted that additives have also been shown to act as crystal nucleation sites to promote crystallinity or control crystal size.^63,64^ In this case, it appears that the former effect was more dominant. It was also found that the crystallinity of the printed samples measured from the first heating curve was slightly greater than the crystallinity obtained for their corresponding filaments cooled in DSC. This was attributed to the processing effect, where shear forces and extensional flows during printing could orient the polymer chains and subsequently promote nucleation and partial crystallization.^65^ The higher Δ*H*_c_ values compared with Δ*H*_m_ of filaments can be attributed to the inherent kinetic asymmetry between crystallization and melting in semicrystalline EVA composites. During cooling, EM shell interfaces may promote heterogeneous nucleation of EVA crystals, increasing the apparent crystallization enthalpy. During reheating, however, imperfect or constrained crystals may melt over a broad temperature range or undergo reorganization, resulting in a lower apparent melting enthalpy. Similar discrepancies between Δ*H*_c_ and Δ*H*_m_ have been reported in semicrystalline copolymers and filled polymer systems.^66,67^

**Table 3:** DSC Data of EVA and EVA/EM Materials in Both Filament and 3D Printed Forms

	heating (endo peak)		cooling (exo peak)		crystallization
specimen	Δ*H*_m_(J/g)	*T*_m_ (°C)	Δ*H*_c_(J/g)	*T*_c_ (°C)	*X*_c, EVA_ (%)
second heating for filament samples					
EVA	–64.66	89.86	76.60	71.00	23.33
EVA/EMs (99/1)	–65.26	89.05	75.73	69.91	23.79
EVA/EMs (95/5)	–62.47	89.21	77.21	69.39	23.73
EVA/EMs (90/10)	–50.39	89.09	77.20	69.42	20.21
first heating for 3D printed samples					
printed EVA	–74.37	89.30	65.48	71.92	26.84
printed EVA/EMs (99/1)	–74.08	88.37	63.57	71.03	27.00
printed EVA/EMs (95/5)	–70.98	88.87	60.63	70.02	26.96
printed EVA/EMs (90/10)	–58.59	88.71	52.93	70.00	23.49

## 4 Conclusions

This work reported the structure formation and mechanical properties of FFF 3D printed EVA foams with tunable densities spanning from 0.903 to 0.224 g/cm^3^. Foamable EVA/EM filaments containing 0–10 wt % EM were extruded and subjected to 3D printing, in which the exposure to heat during printing brought about the inflation of expandable microspheres and thus created microcellular structures. It was found that the process window lies within an optimized range of temperature and flow rate (residence time and feed-in rate) where a printable and foamable object with acceptable shape fidelity and tunable density can be obtained. The extreme combination of high/low temperature and flow rate resulted in issues like unprintability, under-extrusion, overextrusion, or foam shrinkage (Figure S4). The viable print temperature range hinges upon the ratio of EMs, such that a higher loading pushes the temperature window toward lower values. Overall, the foam density revealed an optimal correlation with temperature, reaching the feasible low-end density at 230 °C, regardless of EM content. The analyses depicted the formation of homogeneous microstructures while the bulk density decreased, cell density rose, and cell size marginally fell with EM content.

The mechanical behavior in tension mode showed a decline in critical parameters, including Young’s modulus and strength, as the density dropped proportionally upon adding more EM content. However, it was fascinating to find that the decay rate of Young’s modulus and tensile strength with density reduction was significantly lower, compared to conventional foams, which was attributed to the reinforcing effect of acrylonitrile copolymer EM shell material.

Furthermore, the effects of print pattern and strain rate on the mechanical properties of EVA foams were investigated. It was found that the tensile modulus and strength of perpendicular samples were either equal or only marginally lower than those of parallel samples, indicating that excellent bonding between paths was created in the samples. The response sensitivities of perpendicular and parallel samples to strain rate were also found to be overall similar. Of all three, modulus exhibited the most significant increase in response to strain rate, while strength rose mildly and elongation at break dropped moderately.

Strain hardening over cyclic compressive stress was observed for the solid EVA samples. The occurrence of a strain softening effect as a stark contrast to strain hardening was also reported in low-density foams, attributed to the softening effect of incorporated microspheres at their high loading of 10 wt %. The nonisothermal thermal analyses of the EVA/EM filaments and 3D printed objects showed that EM had a minor impact on the crystallinity of EVA, even though 3D printing caused a slight increment in this characteristic. The findings of this work suggest a novel approach to reduce the density of EVA during printing without the need for conventional secondary procedures while obtaining a mechanically robust structure suitable for various potential applications.

## Supplementary Material









## Notes

Sliced CAD models of the printed parts along with their dimensions and print path directions; SEM micrographs of EMs before and after expansion; selected digital photos from optimizing 3D printed EVA/EMs foams and neat EVA; illustration of processing window for 3D printed EVA/EM foams, revealing the interplay between print temperature and flow rate; detailed information about stress-time response in cyclic compression; and compression stress over time in cyclic loading-unloading of 3D printed EVA and EVA/EM foams (PDF)
  FFF 3D printing of EVA foams (MP4)
  Twisting and bending of the 3D printed foams consisting of 5% EMs (MP4)
  Cyclic compressive stress-strain for 3D printed foams consisting of 5% EMs (MP4)
