# Supplementary material for: Unveiling the Printability and Performance of 3D Printed Ethylene-Vinyl Acetate Stretchable Foams
Source: ACS Appl Mater Interfaces. 2026 Jul 16;18(29):39895–907. doi: 10.1021/acsami.6c10093 (PMC13425563; doi:10.1021/acsami.6c10093)
Supplement: Supplementary file 1 [file am6c10093_si_001.pdf]

Supporting Information

Unveiling the printability and performance of 3D printed ethylene-vinyl acetate stretchable foams

Nariman Rajabifar, Amir Ameli*

Department of Plastics Engineering, University of Massachusetts Lowell, 1 University Avenue, Lowell, MA 01854, United States

Corresponding author: amir_ameli@uml.edu

CONTENT:

- Supporting text and figures
- Supporting movies SM1-SM3
- Supporting references

SUPPORTING TEXT & FIGURES

Supporting Section 1. FFF 3D printing of EVA foams

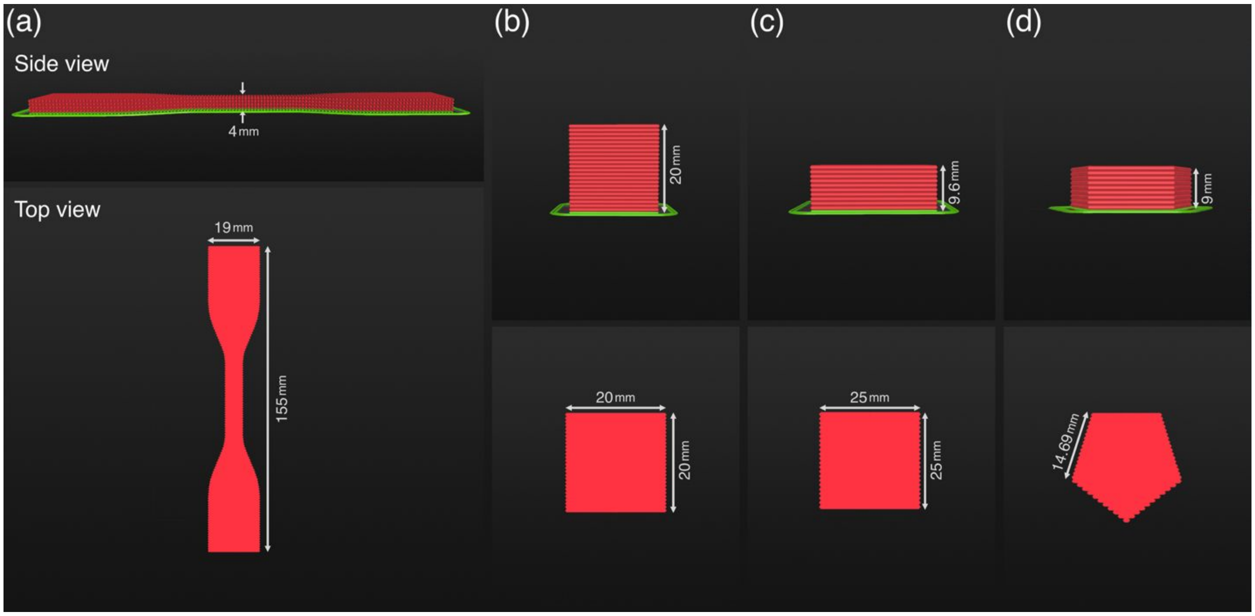

Figure S1. The sliced CAD models of the printed parts along with their dimensions and print path direction. (a) ASTM type IV tensile specimen showing print direction parallel to the tensile loading direction. Tensile specimens were also printed with transverse print direction, providing rater print direction perpendicular to loading direction. (b) Cubic compression testing sample. (c) Compression-set sample. (d) Pentagon object printed at three nominal heights of 6, 9 and 12 mm.

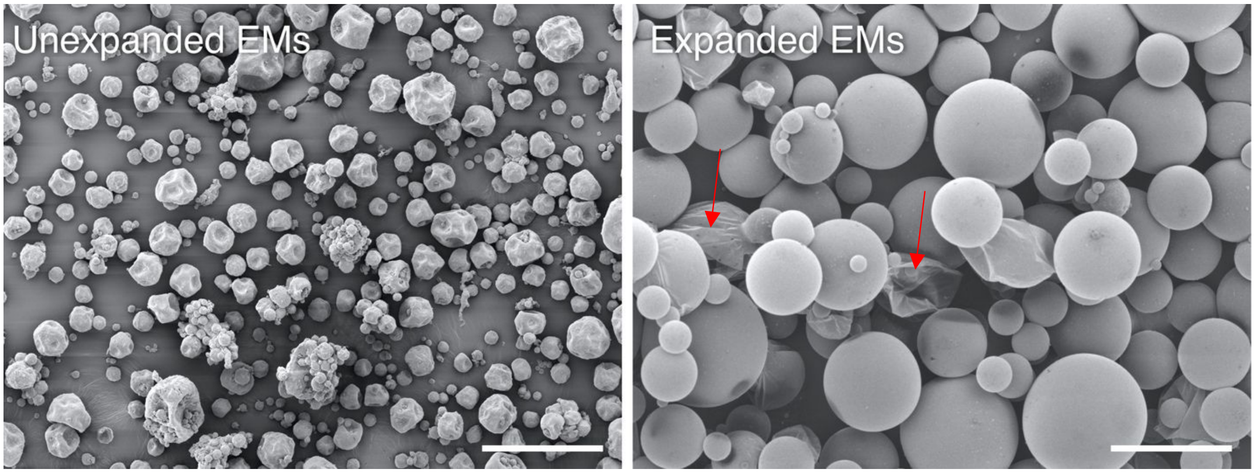

Figure S2. The microstructure of EMs before and after expansion. The expansion obtained freely in an oven from 1 g EMs in an aluminum cup heated to 180 ˚C for half a minute to demonstrate the change of EM morphology. The red arrows show decayed EMs. As the expansion temperature or time increases, the number of these decayed EMs rises.

The EMs used in this work contain an acrylonitrile shell, entrapping hydrocarbons, and have an activation start range of 169–189 ˚C. The composition of Nouryon 980MB100 comprises 65% hydrocarbon and 35% EVA as the carrier. Before activation, the EM particles are spherical (**Figure**). When EMs are exposed to high enough heat, the entrapped volatile turns into a gas and prompts the expansion of the softened acrylonitrile shell. This onset occurs when the EM internal pressure reaches a level that starts deforming the shell elastically[1]. The expansion continues within the temperature range of 169–189 ˚C to 209–229 °C. The strain hardening of the shell during the expansion prevents premature shell rupture.

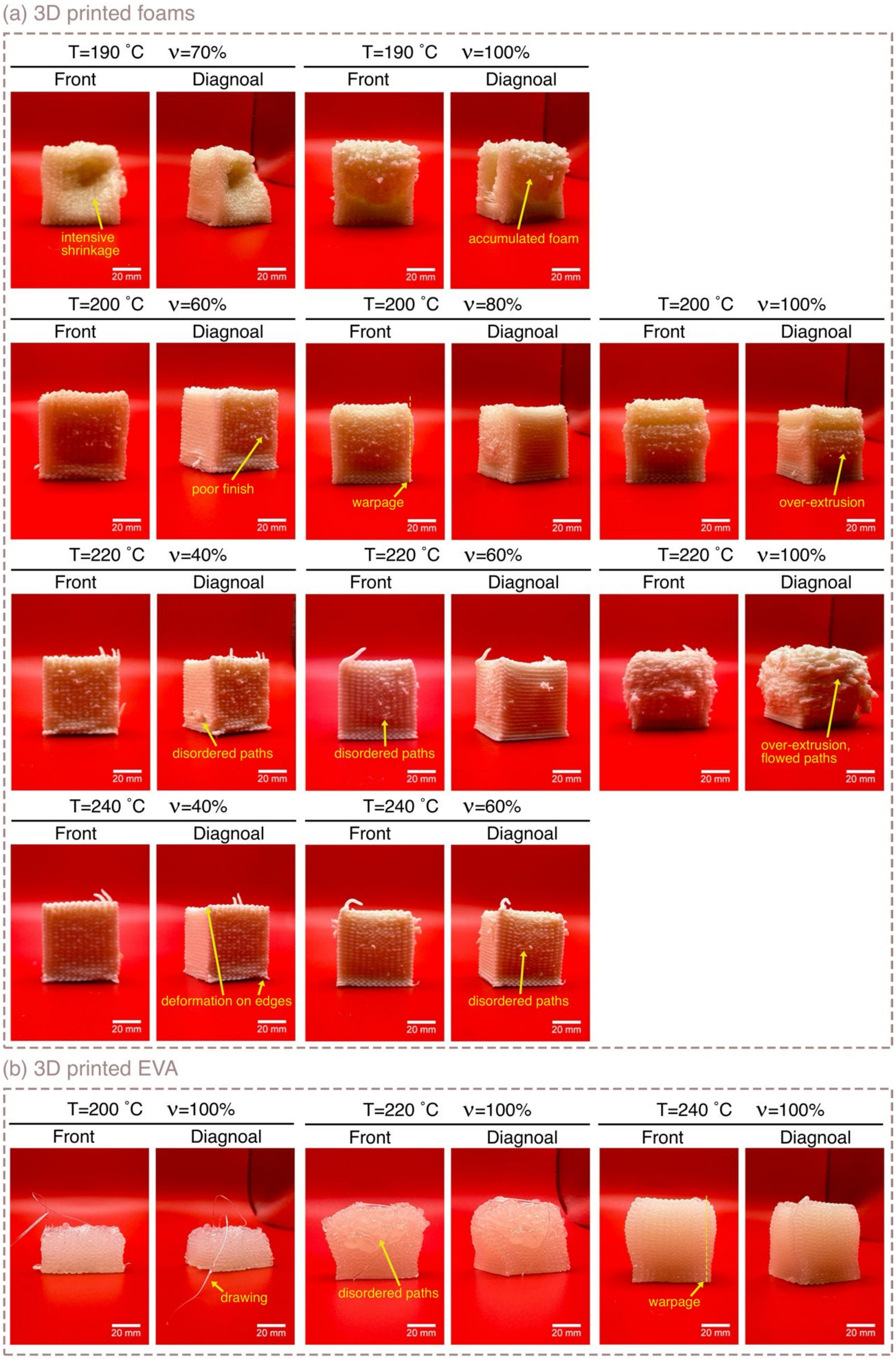
(a) 3D printed foams
T=190 ˚C ν=70%
Front
Diagnoal
intensive shrinkage
T=190 ˚C ν=100%
Front
Diagnoal
accumulated foam
T=200 ˚C ν=60%
Front
Diagnoal
poor finish
T=200 ˚C ν=80%
Front
Diagnoal
warpage
T=200 ˚C ν=100%
Front
Diagnoal
over-extrusion
T=220 ˚C ν=40%
Front
Diagnoal
disordered paths
T=220 ˚C ν=60%
Front
Diagnoal
disordered paths
T=220 ˚C ν=100%
Front
Diagnoal
over-extrusion, flowed paths
T=240 ˚C ν=40%
Front
Diagnoal
deformation on edges
T=240 ˚C ν=60%
Front
Diagnoal
disordered paths
(b) 3D printed EVA
T=200 ˚C ν=100%
Front
Diagnoal
drawing
T=220 ˚C ν=100%
Front
Diagnoal
disordered paths
T=240 ˚C ν=100%
Front
Diagnoal
warpage
20 mm

Figure S3. Selected digital photos from optimizing 3D printed EVA/EMs foams and neat EVA. (a) The effect of temperature and flow rate on the shape fidelity of the EVA/EMs 99/1 specimen. (b) The effect of temperature on the shape fidelity of neat EVA at 100% flow rate due to the lack of foam content.

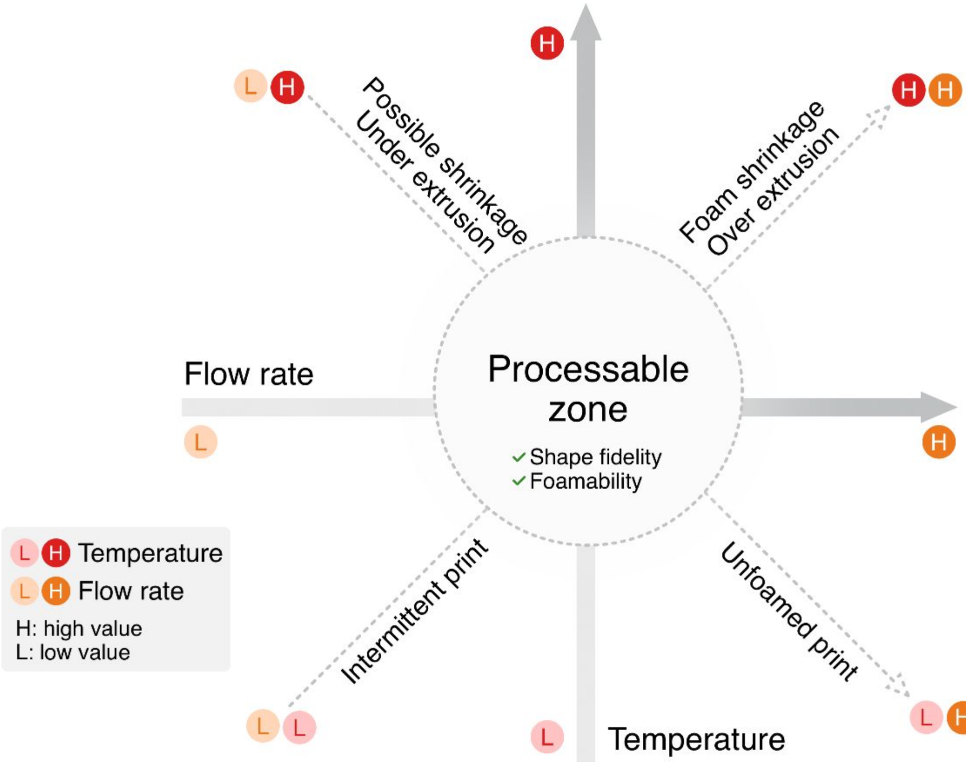

Figure S4. An illustration of processing window for 3D printed EVA/EMs foams, revealing the interplay between print temperature and flow rate. A sweat spot of processable foams with acceptable shape fidelity and tunable density lies in moderate temperatures and flow rates. Processing challenges or part defects associated with the use of extreme levels of temperature and flow rate are identified.

The combination of temperature and flow rate plays a pivotal role in crafting well-designed objects for foam 3D printing owing to the heat-responsiveness of the EMs (**Figure S4**). Trials with different nozzle temperatures and printing speeds showed that printing below 190 ^{o}C is inconsistent. A collection of processing-related deficiencies came up in lieu of a non-disrupted 3D path, namely severe shrinkage, warpage, flowed path, and disordered foaming.

For 3D printing of neat EVA, the flow rate was set at 100% due to the lack of any foamable content. In fact, the dimension of the solid material remains invariant before and after extrusion on account of depositing a similar volume. Having said that, obtaining a well-deposited object in FFF 3D printing still requires a sweet spot in the range of temperature and printing speed to control shape fidelity[2,3]. In this work, the set temperature was at 230 ^{o}C, unaltered between foam and solid polymer. Relatively low viscosity of EVA at 230 ^{o}C resulted in a series of defects, including drawing, warpage, and disordered paths during printing. To overcome this issue, the printing speed for neat EVA was found to be proper at 4 mm/s, which is six units below the considered parameter for EVA/EMs foams. Other parameters remained similar. The thermogravimetric test did not show any sign of degradation for solid EVA.

Supporting Section 2. Stress-time response in cyclic compression

An interesting aspect of the cyclic compression test lies in observing the change of viscoelastic behavior of the material (in this work, EVA and EVA foams) after multiple load-unloading stresses. The evolution of peak stress values over multiple cycles reveals whether the 3D printed samples undergo the Mullins effect, also known as cyclic softening by decreasing peak stress, or strain hardening by showing an upturn behavior[4–6].

For 3D printed EVA, the strain hardening was observed where the successive cycles revealed higher stress. This change is more prominent in about first ten cycles. Although the occurrence of strain-induced crystallization could explain such an incident, we believe this phenomenon may not account for it since our observation is in compression mode. In tension testing, however, strain-induced crystallization is more common due to the effect of stretching force on rearranging the polymer chains. Instead, our result can most likely be justified by the structural reinforcement in loading. To validate this hypothesis, the cyclic compression for 3D printed EVA with a larger nozzle diameter ($\phi = 1\ mm$) was performed. The stress-time response exhibits the same trend as previously visited by the typical nozzle used in the study, albeit the peak of stress in all stress-strain graphs is slightly lower. In fact, the larger diameter causes the formation of a larger inter-path gap, a structural defect, which results in lower stress values[7–9]. When exposed to a compression force, these gaps essentially reduce the densification effect, therefore obtaining relatively lower stress that the polymer can withstand. Over cycles, these gaps could potentially reduce in size and increase the bulk resistance of the material for deformation, which result in an increased peak stress. Some have tied this behavior with viscoelastic stiffening when insufficient recovery time between cycles leads to loading the sample at a higher strain rate, which can appear as stiffening. Although not common, such behavior can be a pro for applications requiring increased resistance to repeated loading, such as protective gear, since it becomes more supportive after an initial break-in period[10–12].

The behavior of 3D printed foams with 99/1 and 95/5 compositions is similar to EVA, although the severity of strain hardening is reduced. The repeated compression causes cell walls to collapse and make contact with one another, effectively increasing the density of the material in localized regions. However, when 10 wt% EM is used, a reverse behavior is observed. Rather than stiffening, EVA/EMs at the 90/10 ratio follows the Mullins effect, which demonstrates the occurrence of softening. Under significant deformations (50% strain here), the EMs present within the EVA can undergo interfacial debonding, cracking, severe permanent deformation, or cause localized yielding of EVA near the interface. All these factors can reduce the load-bearing capability of the EVA/EMs foams at subsequent loading and thus cause strain softening.

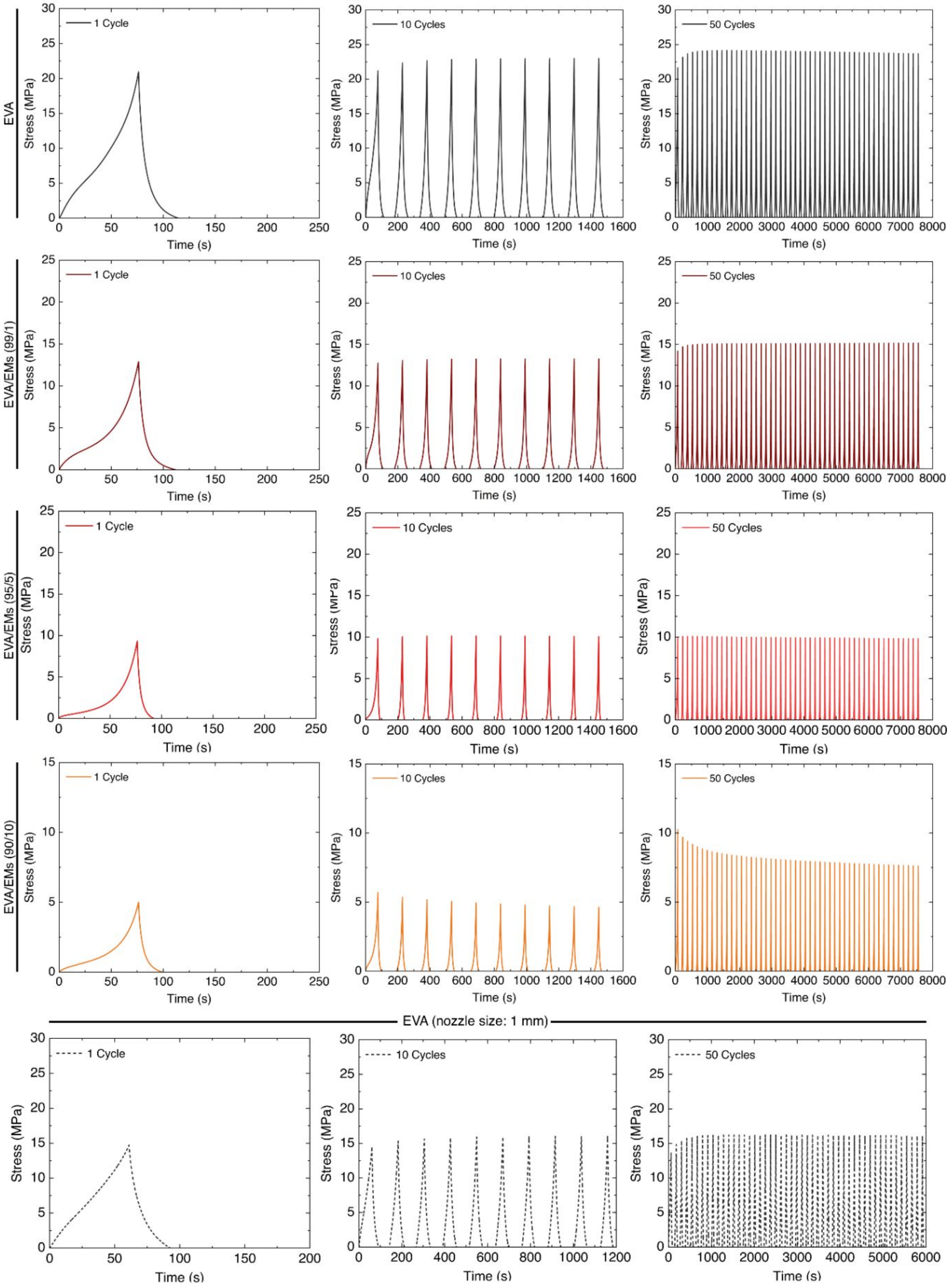

Figure S5. Compression stress release over time in cyclic stress-strain for 3D printed EVA, EVA foam, and EVA with 1 mm nozzle.

SUPPORTING MOVIES

Movie SM1.

FFF 3D printing of EVA foams.

Movie SM2.

The twisting and bending of the 3D printed foams consisting of 5% EMs.

Movie SM3.

Cyclic compressive stress-strain for 3D printed foams consisting of 5% EMs.

SUPPORTING REFERENCES

(1) Kawaguchi, Y.; Oishi, T. Synthesis and Properties of Thermoplastic Expandable Microspheres: The Relation between Crosslinking Density and Expandable Property. *J. Appl. Polym. Sci.* **2004**, *93* (2), 505–512. https://doi.org/10.1002/app.20460.

(2) Ferretti, P.; Leon-Cardenas, C.; Santi, G. M.; Sali, M.; Ciotti, E.; Frizziero, L.; Donnici, G.; Liverani, A. Relationship between FDM 3D Printing Parameters Study: Parameter Optimization for Lower Defects. *Polymers* **2021**, *13* (13), 2190. https://doi.org/10.3390/polym13132190.

(3) Kristiawan, R. B.; Imaduddin, F.; Ariawan, D.; Ubaidillah; Arifin, Z. A Review on the Fused Deposition Modeling (FDM) 3D Printing: Filament Processing, Materials, and Printing Parameters. *Open Eng.* **2021**, *11* (1), 639–649. https://doi.org/10.1515/eng-2021-0063.

(4) Diani, J.; Fayolle, B.; Gilormini, P. A Review on the Mullins Effect. *Eur. Polym. J.* **2009**, *45* (3), 601–612. https://doi.org/10.1016/j.eurpolymj.2008.11.017.

(5) Bueche, F. Molecular Basis for the Mullins Effect. *J. Appl. Polym. Sci.* **1960**, *4* (10), 107–114. https://doi.org/10.1002/app.1960.070041017.

(6) Lee, D.-K.; Hur, O.; Kim, E.; Kang, B.-H.; Kang, S. H.; Min, K.; Park, S.-H. Unveiling Unexpected Mechanical Softening/Stiffening in Carbon Nanotube Composites under Cyclic Deformation: Experiments and Predictive Modeling. *Adv. Compos. Hybrid Mater.* **2025**, *8* (2), 194. https://doi.org/10.1007/s42114-025-01291-4.

(7) Kedare, P. K.; Khan, S. A.; Kumar, H. 3D Printer Nozzle Design and Its Parameters: A Systematic Review. In *Proceedings of International Conference in Mechanical and Energy Technology: ICMET 2019, India*; Yadav, S., Singh, D. B., Arora, P. K., Kumar, H., Eds.; Springer: Singapore, 2020; pp 777–785. https://doi.org/10.1007/978-981-15-2647-3_73.

(8) Tezel, T.; Kovan, V. Determination of Optimum Production Parameters for 3D Printers Based on Nozzle Diameter. *Rapid Prototyp. J.* **2021**, *28* (1), 185–194. https://doi.org/10.1108/RPJ-08-2020-0185.

(9) Tlegenov, Y.; Hong, G. S.; Lu, W. F. Nozzle Condition Monitoring in 3D Printing. *Robot. Comput.-Integr. Manuf.* **2018**, *54*, 45–55. https://doi.org/10.1016/j.rcim.2018.05.010.

(10) Schrauwen, B. A. G.; Janssen, R. P. M.; Govaert, L. E.; Meijer, H. E. H. Intrinsic Deformation Behavior of Semicrystalline Polymers. *Macromolecules* **2004**, *37* (16), 6069–6078. https://doi.org/10.1021/ma035279t.

(11) Lin, P.; Cheng, S.; Wang, S.-Q. Strain Hardening During Uniaxial Compression of Polymer Glasses. *ACS Macro Lett.* **2014**, *3* (8), 784–787. https://doi.org/10.1021/mz5004129.

(12) Salem, A. A.; Kalidindi, S. R.; Doherty, R. D. Strain Hardening Regimes and Microstructure Evolution during Large Strain Compression of High Purity Titanium. *Scr. Mater.* **2002**, *46* (6), 419–423. https://doi.org/10.1016/S1359-6462(02)00005-2.
